# Intra-abdominal pressure in third trimester pregnancy complicated by acute pancreatitis: an observational study

**DOI:** 10.1186/s12884-015-0651-8

**Published:** 2015-09-22

**Authors:** Liqun Sun, Weiqin Li, Fuxi Sun, Yanxia Geng, Zhihui Tong, Jieshou Li

**Affiliations:** Department of Critical Care Medicine, The Second Affiliated Hospital of Nanjing Medical University, Nanjing, China; Institute of General Surgery, Jinling Hospital, Medical School of Nanjing University, Nanjing, China; Department of Intensive Care Unit, Jiangsu Province Chinese Medicine Hospital, Nanjing, China

## Abstract

**Background:**

It is known that intra-abdominal hypertension has high morbidity in acute pancreatitis and has detrimental effects on patients. For third trimester pregnancy complicated by acute pancreatitis, the intra-abdominal pressure may have its own characteristic. This article will discuss this clinical scenario.

**Methods:**

This observational study in a cohort group was performed in the surgical intensive care unit of a tertiary hospital. Medical records were reviewed from each acute pancreatitis exactly in third trimester pregnancy. The main statistical methods were Mann–Whitney *U* test and bivariate Pearson correlation analysis.

**Results:**

During the study interval, there were totally 17 pregnancies complicated by acute pancreatitis in the third trimester. All cases with moderate or severe acute pancreatitis had intra-abdominal hypertension of mean value of 16.7 mm Hg (range, 12.9–22.0 mm Hg). The intra-abdominal pressure had significant correlation with APACHE II score (*r* = 0.7456, *p* = 0.0006), while a negative correlation was showed with the umbilical artery pH value and with 1-min Apgar score (*r* = −0.8232, *p* = 0.0005; *r* = −0.7465, *p* = 0.0034; respectively). The intra-abdominal pressure of those with live infants was lower than that of those with dead ones (13.78 ± 2.554 vs. 19.84 ± 1.695, *p* = 0.0019).

**Conclusions:**

The incidence of intra-abdominal hypertension seems higher in moderate or severe acute pancreatitis in third trimester pregnancy than the non-pregnant cases but there’s no significance in this study. Acute elevated intra-abdominal pressure accounts for great association with mother’s serious scenario and fetal mortality.

## Background

Elevated intra-abdominal pressure (IAP) is commonly present in critically ill patients. Intra-abdominal hypertension (IAH) has detrimental effects on haemodynamics, respiratory and renal function and may eventually lead to multi-organ failure [1]. It is reported that the mean intra-abdominal pressure at the time of admission is an independent predictor of mortality. The mortality in those patients with IAH on admission is 29.8 % versus 18.6 % in those without IAH [2]. In acute pancreatitis (AP), IAH may occur due to interstitial and retroperitoneal inflammatory swelling. The incidence of IAH is approximately 60-80 % in severe acute pancreatitis (SAP) [3].

AP is rare in pregnancy with the incidence ranging from 1:1 000 to 1:10 000 [4, 5]. It usually occurs during the third trimester [6]. Although AP is a rare event in pregnancy, its’ consequences can greatly bring the risk of maternal and fetal morbidity and even mortality [5]. As such more specialists focus on AP in pregnancy. However, to our knowledge, the IAP in pregnant women complicated by AP in the third trimester has seldom been studied.

In this study, we reviewed medical records of AP in third trimester pregnancy in order to investigate the incidence of IAH in these populations and to search for a possible correlation between IAP and maternal-fetal outcomes.

## Methods

This was an observational study over an 8-year period from January 2006 to December 2013 and was granted ethical approval by the Institutional Review Board of Jinling Hospital, a university referral hospital. Informed consent and consent to publish was obtained from all patients for being included in the study. Medical records were reviewed for every third trimester pregnant woman who was transferred into intensive care unit for treatment of AP. Those women who received tocolysis were followed up until delivery.

The Data that were collected included demographic information regarding past medical history, maternal age, and gestational age as well as laboratory parameters, imaging findings, acute physiology and chronic health evaluation (APACHE) II scores on admission, and IAP prior to the delivery. We also recorded maternal outcomes such as local and systemic complications, organ failure, intensive care unit length of stay (ICU LOS), and fetal outcomes such as umbilical artery pH value and 1-min Apgar score of live-born infant, as well as perinatal death.

According to the Atlanta criteria [7], AP is defined as abdominal pain with serum lipase activity (or amylase activity) three times greater than the upper limit of normal and diagnosis is aided by transabdominal ultrasonography before delivery or contrast-enhanced computed tomography after delivery. The severity is classified as mild, moderate or severe. Mild acute pancreatitis (MAP) has no organ failure, local or systemic complications and usually resolves in the first week. Moderately severe acute pancreatitis (MSAP) is defined by the presence of transient organ failure, local complications or exacerbation of co-morbid disease. SAP is defined by persistent organ failure (organ failure >48 h). Organ failure is defined as a score of 2 or more using the modified Marshall scoring system. Local complications are peripancreatic fluid collections, pancreatic and peripancreatic necrosis (sterile or infected), pseudocyst and walled-off necrosis (sterile or infected). Exacerbation of pre-existing co-morbidity precipitated by the acute pancreatitis is defined as a systemic complication.

IAH is defined by a sustained or repeated pathological elevation in IAP ≥ 12 mmHg [8]. It is graded asfollows: Grade I: IAP 12–15 mmHg; Grade II: IAP 16–20 mmHg; Grade III: IAP 21–25 mmHg; Grade IV: IAP 25 mmHg. Abdominal compartment syndrome (ACS) is defined by a sustained IAP above 20 mm Hg with new-onset or progressive organ failure [8]. Twenty five ml of saline was injected into the bladder after emptying. Then the pressure was measured indirectly through the bladder at end-expiration in the supine position with 10° left lateral tilt, ensuring that abdominal muscle contractions and uterine contraction were absent and the transducer was zeroed at the level of the mid-axillary line [9].

Upon admission each case received standard monitoring and treatment in order to maintain normal vital signs. Management included but was not limited to: fluid resuscitation, ultrasound examination for both the mother and the fetus, early fasting and then enteral feeding through nasojejunal tube, gentle laxatives, and consultation from obstetricians. Besides those mentioned above, each woman had a transurethral bladder catheter placed and received IAP measurement every 4 h until delivery or the third consecutive normal value. The value of IAP which was documented in the data set was the average of two consecutive maximum values.

The correlation between IAP and maternal-fetal outcomes was explored by comparing the IAP and maternal demographics [age, GAO, gestational age delivered (GAD)] and outcomes of mother (APACHE II score, ICU LOS) and fetus (umbilical artery pH value and Apgar score at 1 min). We also observed the correlation between the IAP of women carrying live infants and that of those women with non-viable pregnancies or stillborn infants.

Cause is ascertained based on the history, ultrasound and laboratory studies [5]. Biliary pancreatitis is defined as the presence of gallstones or sludge in the biliary tree or the gallbladder. Hyperlipidemic pancreatitis is diagnosed when serum triglyceride level is >11.3 mmol/L (1 000 mg/dl). Cases involving alcohol consumption are classified as alcoholic pancreatitis. Acute pancreatitis associated with drugs, postendoscopic retrograde cholangiopancreatography and abdominal surgery are grouoped as ‘other causes’.

Based on the weeks of gestational age of onset (GAO), third trimester is defined as ≥27 weeks [10]. Term pregnancy is defined as delivery between 37 + 0 and 41 + 6 weeks. A sustained decrease in fetal heart rate is indicative of fetal distress. Intrapartum stillbirth is defined as a stillbirth where intrauterine fetal death occurred following the onset of labor but prior to the birth. Neonatal death is defined as the death during the first 4 weeks after the birth [11].

### Statistical analysis

Data were presented as mean ± SD, or median (range) for continuous variables or *n* (%) for categorical data. Comparison between groups was performed by Mann–Whitney *U* test. Pearson’s bivariate correlation analysis was conducted to analyze the associations between IAP and variables. All tests were two-tailed, and *p* < 0.05 was considered significant. Statistical analysis was performed using SPSS16.0 software package (SPSS Inc, Chicago, IL, USA).

## Results

During the study interval, a total of 53 pregnant women with AP were admitted to our hospital, making up 4.35 % of all cases of AP. Seventeen women were in the third trimester. The median maternal age at admission was 28 years (range, 21–35 years) and the mean GAO was 34.86 weeks. The mean time of delivery was 35.94 weeks. The detailed demographic data were shown in Table 1. One patient (5.9 %) had experienced the first attack of MAP in her second trimester of the same pregnancy. Twelve women (70.6 %) were nulliparous, three women (17.6 %) had one child, and one woman had two children. The mean prepregnant body mass index (BMI) of this group was 22.5 Kg/m^2^ (range, 18.8–24.8 Kg/m^2^). Except the estimated weight of fetus and amniotic fluid (preliminary judged by ultrasound), the mean of BMI on addition was 24.9 Kg/m^2^ (range, 21.5–28.1 Kg/m^2^).

**Table 1:** Summary of patients

Grade,	Demographics					Material outcomes				Fetal outcomes			
The highest IAP prior to delivery (mmHg)	Age (years)	GAO (weeks)	GAD (weeks)	G/P	APACHE II Score	Local complications	Systemic complications	Organ system (failure)	ICU LOS (day)	Mode of delivery (time)	Perinatal outcome	Umbilical artery pH value	1-min Apgar scores
<12,													
8.8	25	35.57	39.43	1/0	2	N	-	N	8	SVD	live	7.28	10
10.6	23	30.00	37.29	1/0	3	N	-	N	4	SVD	live	7.31	9
11.8	29	32.71	35.71	1/0	4	N	-	N	3	SVD	live	7.33	9
G-I(12–15)													
12.9	26	33.43	36.29	1/0	6	N	-	T- Res	12	SVD	live	7.29	10
13.2	21	35.29	35.29	1/0	14	N	DM	T- Res	26	CS (D-0)	live	7.13	5
14	33	29.29	30.29	3/1	8	N	-	T- Res	8	IL (D-7)	-	-	-
14	24	39.43	39.43	1/0	14	Peripancreatic Necrosis	-	T- C; P- Res	22	CS (D-0)	live	7.09	5
14.7	28	33.00	33.43	1/0	9	Peripancreatic Necrosis	HT	T- C; P-Res/R	35	CS (D-3)	live	7.06	5
14.7	29	39.71	39.71	3/1	7	pseudocyst	-	P- Res	24	CS (D-0)	live	6.97	2
G-II(16–20),													
16.0	29	34.86	34.86	1/0	10	Peripancreatic Necrosis	-	P- Res	17	CS (D-0)	live	7.06	5
16.9	28	36.86	36.86	1/0	9	Pancreatic Necrosis	-	P-Res/C	31	CS (D-0)	live	7.04	2
17.7	34	35.86	35.86	4/1	21	Pancreatic Necrosis	-	P- Res	10	CS (D-0)	live	7.08	8
18.4	35	29.29	29.29	7/3	8	Peripancreatic Fluid Collections, Pancreatic Necrosis	HBP	P-Res/R	37	CS (D-0)	Neonatal death	-	-
18.4	30	36.43	36.43	3/1	12	Well-off Necrosis	HL/HBP/DM	P-Res/R./C	111	CS (D-0)	Neonatal death	-	-
19.1	28	36.86	36.86	1/0	9	Well-off Necrosis	HBP	P-Res/R./C	51	CS (D-0)	Intrapartum stillbirth	-	-
G-III(20–25),													
21.3	24	38.00	38.00	1/0	18	Pancreatic Necrosis	-	T- R.; P-Res/C	28	CS (D-0)	Intrauterine fetal death	-	-
22..0	28	36.00	36.00	1/0	22	Well-off Necrosis	-	T- C; P-Res	14	CS (D-0)	Intrauterine fetal death	-	-

C, cardiovascular; CS, cesarean section; DM: diabetes mellitus; GAD, gestational age delivered; GAO, gestational age of onset; HBP: high blood pressure; HL: hyperlipidemia; HT: Hypothyroidism; IL, induced labor; LOS, length of stay; P-: permanent; Res, respiratory; R, renal; SVD, spontaneous vaginal delivery. T-: transient

The main etiology included biliary disease and hyperlipidemia (Table 2). Asymptomatic abnormalities were found in 10 women (58.8 %) by abdominal ultrasound examination. Seven of them were found to have gallstones and 3 of them have sludge. As 3 of those with asymptomatic abnormalities simultaneously had severe hypertriglyceridemia, they were classified as hyperlipidemic pancreatitis.

**Table 2:** Etiologic diagnosis of acute pancreatic in pregnancy

Etiologic diagnosis	MAP (*n* = 3)	MSAP (*n* = 3)	SAP (*n* = 11)	Total cases (*n* = 17)
Biliary pancreatitis	2	1	4	7 (41.2 %)
Hyperlipidemic pancreatitis		1	7	8 (47.1 %)
Simultaneous biliary abnormalities			3	3 (17.6 %)
Alcoholic pancreatitis	1			1 (5.9 %)
other causes		1		1 (5.9 %)

Normal IAP was detected in three women with MAP. They recovered quickly with conservative treatment and had term infants born spontaneously. In contrast, IAH developed in 14 (82.4 %) pregnant women with mean value of 16.7 mm Hg (range, 12.9–22.0 mm Hg). They were diagnosed with MSAP or SAP. That is to say, all the patients with MSAP or SAP manifested some degree of IAH (Table 3). Two women (11.8 %) with SAP developed ACS with the mean IAP of 21.7 mmHg and new-onset organ failure. Organ failure of varying severity was witnessed and was shown in Table 1. Three cases with mild elevated IAP (Grade I) had no local complications. They were diagnosed with MSAP for the transient organ failure (transient respiratory failure). The other 11 women with IAH of grade I or grade II were classified as SAP because of both local complications and permanent organ failure: Six cases (54.5 %) had abscess formation, 4 cases (36.4 %) developed necrosis, and one case (9.1 %) had acute fluid collection.

**Table 3:** IAP in acute pancreatic in pregnancy

Diagnosis	Mean IAP (mmHg)					
	Non-IAH	IAH-Grade I	IAH-Grade II	IAH-Grade III	IAH-Grade VI	IAH-ACS
MAP (*n* = 3)	10.4	-	-	-	-	-
MSAP (*n* = 3)	-	13.4	-	-	-	-
SAP (*n* = 11)	-	14.5 (*n* = 3)	17.8 (*n* = 6)	21.7 (*n* = 2)	-	21.7 (*n* = 2)*

*Two women developed IAH of grade III manifested ACS

Twelve patients (70.6 %) had to undergo prompt caesarean section (CS) for signs of fetal distress and continued clinical instability despite ICU admission. Among those cases, two patients underwent simultaneous surgical debridement and drainage of infected pancreatic necrosis, whereas the other 10 patients underwent percutaneous drainage and/or surgical treatment in the later stages of disease.

The correlation of IAP with age, GAO, GAD was insignificant (*r* = 0.3418, *p* = 0.18; *r* = 0.2950, *p* = 0.25; *r* = 0.1676, *p* = 0.52; respectively). APACHE II score had significant positive correlation with IAP (*r* = 0.7456, *p* = 0.0006). However, IAP had no significant correlation with ICU LOS (*r* = 0.4336, *p* = 0.08). The umbilical artery pH value and Apgar score at 1 min showed a negative correlation with IAP (*r* = −0.8232, *p* = 0.0005; *r* = −0.7465, *p* = 0.0034; respectively).

**Fig. 1:**
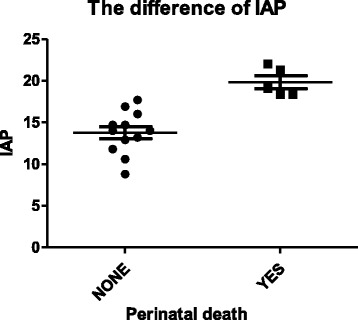
IAP in live birth group and perinatal death group. The IAP of cases with live infants was lower than that of those with dead ones (13.782.55 vs. 19.841.70, *p* = 0.0019)

No maternal death was recorded. Except for one fetal loss due to selectively induced labor, perinatal mortality rate was 31.2 %, consisting of 3 cases of intrauterine fetal demise (including 1 case of intrapartum stillbirth) and 2 cases of neonatal death. The fetal and placental pathology analysis can’t be implemented because of the family’s refusal. The IAP of women carrying live infants was lower than that of those women with non-viable pregnancies (13.78 2.55 vs. 19.84 1.70, *p* = 0.0019) (Fig. 1). There was no significant difference between those two groups in maternal demographics.

## Discussion

As the mortality of patients with IAH is higher than those without IAH, surveillance and early management of IAH is increasingly implemented in these IAH-prone patients. Pregnancy have physiologic progressive IAP elevation usually close to the diagnostic threshold of IAH in the third trimester [12]. Acute intra-abdominal inflammation due to acute pancreatitis developing in the third trimester definitely has an effect on IAP. In this study, IAH was observed in all the third trimester pregnancy diagnosed with MSAP or SAP. The incidence of IAH in pregnancy was 82.4 %, which was higher than our previous study in the non-pregnancy pancreatitis group (around 65 %, 39/60) [13] but there was no significant difference (*p* = 0.24, *χ*^2^ test). The likely reason was the abdominal wall which was already strained by the enlarged uterus had less ability to tolerant any rapid increase of IAP. The difference need to be observed with more samples in each group in the future.

Previous reports showed the main cause of AP in pregnancy was biliary disease [14, 15]. It is generally mild yet it tends to recur [15]. Notably, most severe cases tend to be hyperlipidemic pancreatitis [5, 16]. Normal pregnancy is known to have physiological hyperlipidemia. It is thought to represent a generalized increase in substrate mobilization both for the placenta and the growing fetus. Clinically significant hyperlipidemia characteristically occurs in the third trimester when lipid clearance is outpaced by lipid synthesis and release. Pancratitis, only with triglyceride levels above 11.3 mmol/L, is defined as hyperlipidemic pancreatitis [17]. In the present study, nearly 50 % patients (8/17) met the diagnostic criteria of hyperlipidemic pancreatitis. Except one patient with MSAP, the other seven patients were diagnosed as SAP. Severe obesity is one kind of reason of increased IAP. In this group the mean BMI on addition was near the ideal range of pregnant BMI according to the 2009 Institute of Medicine guidelines [18].

Table 3 shows the distribution of IAH in different classifications of AP in pregnancy. All the patients with MSAP or SAP manifested different grades of IAH (100 %), including two cases manifesting ACS. Significantly increased IAP could reduce intra-abdominal organ perfusion and contribute to deterioration in organ function [2, 3]. Histological and ultra-structural analysis of the pancreas in a porcine model of IAH showed that IAH might worsen the pancreatitis [19]. Similarly, both SAP and IAH had been demonstrated remarkable negative effects on systematic hemodynamics, oxygenation and organ function in porcine models [20]. Consistent with these results, in this study, IAP had been showed to significantly correlate with maternal APACHE II score (*r* = 0.7456, *p* = 0.0006). In addition, it was reported that mortality in acute pancreatitis patients with ACS was 49 % versus 11 % without ACS [9]. Fortunately, all women in this study survived, though two cases had ACS. These women might benefit from the prompt and effective decrease of IAP by CS.

In pregnant ewes, increased IAP (10 mm Hg for 30 min followed by 15 mm Hg for 30 min) caused reduced uterine blood flow and fetal hypoxemia [21]. Similarly, in our study, 1-min Apgar score and umbilical cord artery pH, as the indexes of hypoxic and ischemic injuries of the fetus [22], were negatively correlated with IAP (*r* = −0.7465, *p* = 0.0034; *r* = −8232, *p* = 0.0005; respectively). Furthermore, the IAP of cases with live infants was significant lower than that of those patients with non-viable pregnancies. A particularly high perinatal mortality of nearly 30 % was observed in this study. Cases with SAP manifesting severe IAH had to undergo termination of pregnancy and/or pancreatic debridement for irreversible signs of fetal distress or continued instability of the mother after the admission.

Recent data suggest that the adverse effects of elevated IAP can occur at much lower levels than previously thought [23]. In this study, devastating consequences to both mother and fetus occurred at an IAP of less than 20 mm Hg, a value which was common and well tolerated in non-pregnant patients with SAP [1, 24]. Unfortunately, intrauterine fetal death occurred in three cases with IAP of more than 19 mm Hg. Therefore, for pregnant women with AP in the third trimester, continued surveillance and early management of IAH as soon as IAP higher than 15 mm Hg may be beneficial to not only protect the mother from further detrimental effects, but also to decrease perinatal mortality.

The general strategies suggested to lower IAP include evacuating intra-luminal contents, evacuating intra-abdominal space occupying lesions, improving abdominal wall compliance, optimizing fluid administration, optimizing systemic and regional tissue perfusion, or as a last resort, decompressive laparotomy [25]. Considering that the expanding uterus in the last trimester occupies most of the intra-abdominal space, the most prompt and effective means to decrease IAP may be the delivery of the fetus (via CS) when the IAP is persistently elevated and a threat to maternal/fetal health. Delivery of the fetus can early avoid excessive negative stimulation to fetus by the acute raised IAP, and might decrease the perinatal mortality. Further case–control studies are needed to confirm these associations. CS is prompt and effective in reducing the intra-abdominal volume as well as moving fetus away from the severe physiologic derangements of the mother. As CS is not a normal delivery method, its indication should be explored further. In the present study, most women with SAP required delivery of the fetus. Even with moderate IAH, CS is the preferred option for delivery. However, it is unfortunate that the IAP of mothers post-delivery has not been monitored regularly and the effect of CS on IAP could not be further qualified. Another limitation of our study is that the abdominal perfusion pressure (mean arterial pressure minus intra-abdominal pressure) was not analyzed which would be an important index of the effects of IAH on organ perfusion. Further studies focused on utero-placental blood supply will be beneficial for the improvement of perinatal mortality.

## Conclusions

The incidence of IAH seems higher in AP in third trimester pregnancy than that of non-pregnant population but there’s no significance in this study. During the course of AP, acute elevated IAP correlates with the severity of mother’s illness and fetal mortality. Hence serial IAP monitoring is of importance for such patients. The IAH threshold for the intervention should be lower than that of general critically ill patients. Due to the sensitivity of the fetus to increased IAP, and the altered maternal physiology in third trimester pregnancy, delivery of the fetus via CS could be a prompt and effective means to protect both mother and fetus in women with severe pancreatitis. As CS is not in accord with pathology, its indication for this kind of populations should be explored further.

## Abbreviations

ACS: Abdominal compartment syndrome
AP: Acute pancreatitis
APACHE: Acute physiology and chronic health evaluation
GAD: Gestational age delivered
GAO: Gestational age of onset
IAH: Intra-abdominal hypertension
IAP: Intra-abdominal pressure
ICU LOS: Intensive care unit length of stay
IL: Induced labor
MAP: Mild acute pancreatitis
MSAP: Moderately severe acute pancreatitis
SAP: Severe acute pancreatitis
